# Developing a Consensus-based Definition of “Kokoro-no Care” or Mental Health Services and Psychosocial Support: Drawing from Experiences of Mental Health Professionals Who Responded to the Great East Japan Earthquake

**DOI:** 10.1371/currents.dis.cfcbaf509711641ab5951535851e572e

**Published:** 2015-01-29

**Authors:** Yuriko Suzuki, Maiko Fukasawa, Satomi Nakajima, Tomomi Narisawa, Asano Keiko, Yoshiharu Kim

**Affiliations:** Department of Adult Mental Health, National Center of Neurology and Psychiatry, National Institute of Mental Health, Kodaira, Tokyo, Japan; Department of Adult Mental Health, National Center of Neurology and Psychiatry, National Institute of Mental Health, Kodaira, Tokyo, Japan; Department of Adult Mental Health, National Center of Neurology and Psychiatry, National Institute of Mental Health, Kodaira, Tokyo, Japan; Translational Medical Center, National Center of Neurology and Psychiatry, National Institute of Mental Health, Kodaira, Tokyo, Japan; Graduate School of Musashino University, Koto, Tokyo, Japan; Department of Adult Mental Health, National Center of Neurology and Psychiatry, National Institute of Mental Health, Kodaira, Tokyo, Japan

## Abstract

Objectives: In this survey, we aimed to build consensus and gather opinions on ‘Kokoro-no care’ or mental health services and psychosocial support (MHSPSS) after a disaster, among mental health professionals who engaged in care after the Great East Japan Earthquake. Methods: We recruited mental health professionals who engaged in support activities after the Great East Japan Earthquake, which included local health professionals in the affected areas and members of mental health care teams dispatched from outside (n = 131). Adopting the Delphi process, we proposed a definition of ‘Kokoro-no care’, and asked the participants to rate the appropriateness on a 5-point Likert scale. We also solicited free comments based on the participants’ experiences during the disaster. After Round 1, we presented the summary statistics and comments, and asked the participants to re-rate the definition that had been modified based on their comments. This process was repeated twice, until the consensus criterion of ≥ 80% of the participants scoring ≥ 4 on the statement was fulfilled. Results: In Round 1, 68.7% of the respondents rated the proposed definition ≥ 4 for its appropriateness, and 88.4% did so in Round 2. The comments were grouped into categories (and subcategories) based on those related to the definition in general (Appropriate, Continuum of MHSPSS, Cautions in operation, Alternative categorisation of care components, Whether the care component should be categorised according to the professional involved, Ambiguous use of psychology, and Others), to mental health services (Appropriate, More specification within mental health services, More explicit remarks on mental health services, and Others), and to psychosocial support (Whether the care component should be categorised according to the professional involved, Raising concerns about the terms, and Others), and others. Conclusion: We achieved a consensus on the definition of ‘Kokoro-no care’, and systematically obtained suggestions on the concept, and practical advice on operation, based on the participants’ experiences from the Great East Japan Earthquake. This collective knowledge will serve as reference to prepare and respond to future disasters.

## Introduction

Experiences and knowledge on disaster mental health have been accumulated for several decades since the landmark work ‘When disaster strikes’ 1, and mental health and psychological care components are, nowadays, a part of comprehensive disaster aid activities. However, people have different understandings of the scope of such care, based on the existing theories or their professional backgrounds, which has caused difficulty in providing comprehensive mental health and psychosocial care to the people affected by disaster. A typical example is the consequence after the earthquake and tsunami in the Indian Ocean in December 2004, during which the aid without a shared scope of the care activities reportedly caused confusion by introducing a variety of intervention services based on different theories and disciplines 2. To learn from these lessons, major actors on mental health and psychosocial care after disaster started discussions to build a consensus on the scope of psychological care after disaster, and this effort lead to a publication of guidelines. These guidelines explained the concept and principles of such care, which were called ‘mental health services and psychosocial support’ (MHSPSS) 3.

The guidelines of the Inter-Agency Standing Committee (IASC) describe the scope of MHSPSS as ‘Mental health and psychosocial support include all activities from inside and outside of the affected community, to protect and promote psychosocial wellbeing, or prevent and treat a person with mental illness. In these guidelines, the IASC has chosen to use this term which has overlapping meaning, because these approaches are different, although complementary’ 3. Mental health approach includes psychiatric services, mental health services, and other efforts to facilitate recovery from mental illness. They are typically provided by healthcare professionals, although there are emerging movements of self-help and consumers’ group activities. On the other hand, psychosocial intervention includes any activities that aim to address the psychological and social issues, to reduce stress and promote quality of life. The psychosocial approach can be defined as a collection of non-medical approaches, and has distance from a mental health approach, which tends to overlap with a psychiatric approach 4. Thus, tangible aid, such as food and housing, and community development, along with addressing the psychological needs of the people, can be seen as psychosocial support. Many aid agencies have accepted this scope suggested by the IASC; however, the existing guidelines which cover mental health and psychosocial support continue to lack clarity regarding the definition and scope of such activities 5 ^,^ 6 ^,^ 7.

With reference to Japan, some studies and activity have been reported after major disasters, including the atomic bombing in Nagasaki in 1945 8. In the wake of the Great Hanshin-Awaji Earthquake in 1995, mental health professionals started mental health care immediately after the onset of the disaster, and lay-volunteers poured to the affected areas to offer humanitarian aid. Owing to this helping movement, the concept of ‘Kokoro-no care’, which is now recognized as MHSPSS, was familiarized among mental health professionals, as well as the public 9 ^,^ 10, whereas “Kokoro” refers to a broad concept of mind, thoughts, and emotions in Japanese. Following high-profile disasters, both natural and man-made, such care was offered to the affected people and communities, and the ministry of Health, Welfare and Labour published guidelines for local mental health care activities after a disaster 11. In addition, mental health care teams, called ‘Kokoro-no care teams’, were coordinated and dispatched to the affected areas, in the acute phase, and a disaster mental health centre, called ‘Kokoro-no care centre’, was established to respond to the increased mental health needs in the long-term, recovery-phase. This was also done after the Great East Japan Earthquake 12. This systematic provision of care has been loosely called ‘Kokoro-no care’.

The people in Japan tend to be prejudiced against mental health/illness 13, and mental health literacy, or knowledge on mental health and mental illness, was found low as compared to other countries such as Australia 14 ^,^ 15. This may have originated in the usage of the Japanese word ‘seishin’, that means ‘psychiatry’ or ‘mental health’ in the healthcare field, and also means ‘mind’ or ‘human spirit’ in colloquial terms 16. The latter is also expressed by the word ‘kokoro’, which is a neutral term used more often in daily conversation. To reduce the stigma attached to psychiatry, and to popularize the concept of psychiatry or mental health, a plain and familiar word ‘kokoro’ is now used to express psychiatry or mental health in the healthcare field in Japan. In addition, the term ‘Kokoro-no care’ was widely used to popularize the mental health services during disasters. However, now ‘Kokoro-no care’ is widely used among both professionals and community people without explicitly stating what it is, leading to confusion in the mental health professionals and community people. For healthcare professionals, ‘Kokoro-no care’ typically indicates the mental health services such as identifying and addressing pathological mental health issues; while community people usually expect hearty care, which may not necessarily include specialized or clinical care. Indeed, there is some overlap between these understandings.

The present paper aims to report the process of building consensus on the definition of ‘Kokoro-no care’ after disaster, among mental health professionals who engaged in the provision of care after the Great East Japan Earthquake. Using the MHSPSS framework presented in the IASC guidelines 3 or IFRC manuals 7, we adopted the Delphi process to systematically build consensus and gather comments based on the participants’ experiences of the disaster.

## Methods

**Participants:** We recruited professionals who had engaged in support activities in areas severely affected by the Great East Japan Earthquake that occurred on 11 March, 2011. These areas included Iwate, Miyagi, and Fukushima prefectures, and Sendai city. (Please note that though Sendai city is geographically a part of the Miyagi prefecture, it has an independent administrative body, and has its own mental health service in time of disaster.) With the help of local mental health professionals who served as a focal point to coordinate mental health services at the prefectural/city, mental health and welfare centre, or the department of psychiatry in the universities, we sent invitations to the local health professionals in the affected areas who hosted mental health care teams, and the members of such teams dispatched from outside. We targeted teams that started their activities in March and April 2011, and invited professionals from different backgrounds and positions, including team leaders and coordinators. We also recruited participants from members of the special committee on the Great East Japan Earthquake, the Japanese Society for Traumatic Stress Studies, and psychiatrists from the local psychiatric hospitals of the severely affected areas.

**The Delphi process:** The Delphi process is typically used to build consensus where scientific evidence is not well accumulated 17. Over several rounds, the participants were presented with a statement, and were asked to rate its appropriateness and provide comments. This process allows them to reconsider and change their answers in the following round, and leads to convergence of their opinions. We asked the participants to rate the appropriateness of a proposed definition of ‘Kokoro-no care’ with a 5-point Likert scale (1 =highly inappropriate, 3 = neither appropriate nor inappropriate, and 5 = highly appropriate), with a separate ‘do not know’ option. After each round, the participants received feedback on summary statistics and summary comments, and were asked to rate the appropriateness until agreement was achieved. This process was repeated until the participants reached the consensus criterion, that is, ≥ 80% of the participants scored the statement ≥ 4 (appropriate or highly appropriate). The survey was conducted using the online survey tool ‘Survey Monkey’ (http://jp.surveymonkey.com/).

**Proposed definition:** In Round 1, the proposed definition of ‘Kokoro-no care’, based on the description in the IASC guidelines and IFRC manual^3^^,^ 7, was: ‘Kokoro-no care’ after a disaster comprises two components: mental health service and psychosocial support. Mental health service refers to a set of services aiming to prevent and treat mental health problems, and facilitate recovery from it, with the professional help of psychiatry, psychology, or community health. For example, assessment and prescription by psychiatrists, counselling by healthcare/social work professionals, therapy by psychologists, psycho-education, and awareness-raising activity regarding traumatic reaction and/or other mental health problems. Psychosocial support refers to the other activities aiming to promote general psychosocial well-being. For example, listening, counselling, lecture, recreation, group activities, and relaxation techniques such as massage and foot bathing. These are typically offered by non-mental health professionals, such as education and healthcare personnel, or lay-volunteers. These activities largely overlap and lie on a continuum. However, it is important that care providers are aware of their roles and positions on this continuum.

**Analysis:** For each round, the proportion of each rating score for the proposed definition was calculated. The text in the free comments were analysed using the content analysis method, in which all the comments were assembled and broken into a unit of analysis, which was an episode in the comments. One researcher (YS) classified the units into categories based on the themes, 1) definition in general, 2) mental health services, 3) psychosocial support, and 4) others. Within each category, YS first grouped similar contents into sub-categories, examining the differences and relationships between them. Next, MF independently classified the comments based on the theme within the category. In case of disagreement, a final decision was made after a discussion. The proportions of agreement between the two researchers, on these categorisations, have been presented in the Results section. In this paper, the sub-categories have been described in bold characters, and the quotes have been indicated using single quotation marks.

**Ethical consideration:** This survey protocol was examined and approved by the Institutional Review Board of the National Centre of Neurology and Psychiatry. Before conducting the survey, written informed consent was obtained from the participants.

## Results

**1. Participant characteristics**

A total of 131 people agreed to participate in this survey, and 115 people completed Round 1 (response rate: 87.8%). The basic characteristics, profession, prior experience in working in a disaster area, and the position they were engaged in after the Great East Japan Earthquake, have been presented in Table 1. Note that 31.3% of the respondents were psychiatrists, 25.2% were public health nurses, and 20.0% were psychiatric social workers, and most of the participants were licensed health professionals.

**Table 1. Characteristics of the participants of Round 1 of the survey (n=115):** 

		n	%
Gender			
	Male	51	44.3
	Female	64	55.7
Age (n = 114)			
	20–29	3	2.6
	30–39	35	30.7
	40–49	34	29.8
	50–59	36	31.6
	60–	6	5.3
Profession (multiple answers allowed)			
	Psychiatrists	36	31.3
	Other physicians	1	0.9
	Public health nurses	29	25.2
	Nurses	13	11.3
	Psychiatric social workers	23	20.0
	Clinical psychologists	12	10.4
	Administrators	4	3.5
	Others	4	3.5
The number of disasters for which they worked before the Great East Japan Earthquake			
	0	76	66.1
	1	18	15.6
	2	13	11.3
	3 +	8	7.0
Their position in the support activities after the Great East Japan Earthquake (multiple answers allowed)			
	Experts of the affected area and working as per their routine practice	28	24.3
	Experts of the affected area and providing special support activities	23	20.0
	Experts dispatched from other areas	73	63.5

**2. The Delphi process**

1) Results of Round 1:

In this round, 68.7% of the respondents rated the proposed definition of ‘Kokoro-no care’ ≥ 4 for its appropriateness. The categories and sub-categories of their comments have been presented in Table 2. There was 95.3% agreement between the two researchers, in terms of the sub-categories in Round 1. The comments from 20 participants consisted of 43 units, which were broken down into 23 units on definition in general, 9 units on mental health services, 4 units on psychosocial support, and 6 units on other aspects that were not related to the proposed definition.

**Table 2. The categories and sub-categories of the comments from Round 1 and 2:** 

Categories and sub-categories		n	Units
**Round 1**		20	43
Definition in general		17	23
	Appropriate		3
	Continuum of MHSPSS		5
	Cautions in operation		5
	Alternative categorisation of care components		3
	Whether the care component should be categorised according to the professional involved		3
	Ambiguous use of psychology		3
	Others		1
Mental health services		9	9
	Appropriate		2
	More specification within mental health service		4
	More explicit remark on mental health service		2
	Others		1
Psychosocial support		4	5
	Whether the care component should be categorised according to the professional involved		3
	Others		2
Others		6	6
**Round 2**		9	9
Definition in general		4	4
	Cautions in operation		2
	Alternative categorisation of care components		1
	Others		1
Mental health services		2	2
	Others		2
Psychosocial support		2	2
	Raising concerns about the terms		2
Others		1	1

**a. Definition in general**: The sub-category, **Appropriate**, included comments that generally agreed with the proposed definition, without additional specific opinions. For example, ‘It is easy to understand’. (Psychologist, dispatched)The sub-category **Continuum of MHSPSS** included comments that emphasized on the continuity and complementary nature of mental health services and psychosocial support. Some of the related quotes were as follows:‘Kokoro-no care is more like a continuous spectrum with professional service on one extreme, and non-professional service for anyone on the other; the two components of activities’. (Psychiatrist, dispatched) ‘In the field, other than in psychiatry, counselling is provided in educational and medical settings. In community-salon-meetings, the element of group therapy can be adopted, and thus, it cannot be categorised as proposed’. (Psychiatric social worker/psychologist, local) With reference to** Cautions in operation**, this sub-category included the comments that generally supported the proposed definition, with practical suggestions regarding operation. ‘Professionals need to have perspective of livelihood support, and non-professionals need to have mental health literacy’. (Psychologist, dispatched) ‘In support activities, needs differ depending on the timing, place, and situation. Thus, it is important to take action after assessing the needs’. (Psychiatrist, dispatched)**Alternative categorisation of care components** included alternative statements that would better describe ‘Kokoro-no care’ than the proposed definition, in that there were two axes of mental health service and psychosocial support in one concept. ‘The word “Kokoro-no care” itself is ambiguous, and it causes confusion for both providers and recipients of care. Thus, mental health service and psychosocial support should be separated’. (Psychiatrist, dispatched) ‘The care component should be organized by the passage of time and needs of the affected people’. (Psychologist, dispatched) The sub-category **Whether the care component should be categorised according to the professional involved** included comments that posed questions related to if the care component needs to be separated by the types of providers. For instance: ‘It is too rough to define mental health service, which includes prevention, treatment, and facilitation of recovery that is provided by professionals, as well as other psychosocial support provided by non-professionals’. (Psychologist, dispatched) The** Ambiguous use of psychology** included comments that referred to the ambiguous meaning and expected role of psychology in the context of disaster aid. ‘I cannot judge if the word “psychological” fits in this description for two reasons. Psychological support is a major component of mental health service; on the other hand, psychologists do not possess the expertise required for “psycho”-social care’. (Psychiatrist, inside) The **Others** category included comments such as ‘It is difficult to understand’.

**b. Mental health services:** **Appropriate** included comments that generally agreed with the proposed definition, without any addition. **More specification within mental health services** included comments that suggested that psychiatric rescue, psychiatric care, mental health services, and psychological support should be separated with specific objectives for each. Such comments read as follows: ‘With the expression of “a set of service to aim to prevent and treat mental health problems”, the nuance of relapse prevention for those with existing mental illness is not fully conveyed. Ensuring prescriptions for patients at risk of treatment discontinuation in the aftermath of disaster, and to the time when access to the local health facilities are ensured, is worth being separated as an independent activity’. (Psychiatrist, local) ‘Within mental health services, it is better to explicitly describe psychiatric services for the treatment level, and mental health services for the prevention level. Such clear distinction would help to develop strategies depending on settings’. (Psychiatrist, dispatched) **More explicit remarks on mental health services** included comments that stressed on the component of mental health services, rather than using a broad term like ‘Kokoro-no care’, for the benefit of the affected people and care providers. ‘“Kokoro-no care” is generally ambiguous. Thus, a more straightforward word would be better to describe mental health services’. (Psychiatrist, local) ‘In reality, we provide mental health and welfare services; thus, it is better to explicitly express this service. In the affected areas, with the word “Kokoro-no care”, some people hesitated from or resisted using the service’. (Psychiatric social worker/psychologist, dispatched) **Others** include comments that stated that better organisation of the roles of general physicians, public health nurses, or clinical nurses are needed within mental health services. ‘Not only psychiatrists, but also internal medicine doctors treat people with sleep problems or depression after a disaster. Similarly, the role of public health nurses and clinical nurses should be better organised’. (Public health nurse, local)

**c. Psychosocial support:** In the psychosocial support category, the comments were classified as **Whether the care component should be categorised according to the professional involved**, and **others**. The former included comments as follows: ‘Psychosocial support varies and is provided by people ranging from medical professionals to lay-volunteers’. (Public health nurse, local) ‘I am concerned that psychosocial support is apparently provided by non-professionals in the proposed definition. In the field, professionals also engage in psychosocial care’. (Nurse, dispatched)**Others** included comments that stated that the concept of psychosocial support itself was unclear, and that health professionals and community people had different understandings of psychosocial support.

**d. Others:** The **Others** category included miscellaneous comments that were not related to the proposed definition, for example, their own experiences in the affected area, statements suggesting an unclear understanding of the definition, etc.

For Round 2, these comments were summarized as follows and presented with the summary statistics: Overall, there were many positive opinions that supported the proposed definition. Some opinions emphasized that the expected activities for ‘Kokoro-no care’ depends on the timing, setting, and situation of the care to be provided. The critique included that the contents of ‘Kokoro-no care’ should be classified by the nature of the activities, and not by the professionals involved, and that it is better to emphasize on the continuity or complementary nature of the activities, rather than on its division into mental health services and psychosocial support.

2) Modified definition

To reflect these comments, we modified the proposed definition (modification underlined), and asked the participants to rate its appropriateness in Round 2.

‘Kokoro-no care’ after disaster comprises two components: mental health service and psychosocial support. Mental health service refers to a set of services aiming to prevent and treat mental health problems, and to facilitate recovery from it, with the professional help of psychiatry, psychology, or community health. For example assessment and prescription by psychiatrists, counselling by health/social work professionals, therapy by psychologists, psycho-education, and awareness-raising activities regarding traumatic reactions and/or other mental health problems. Psychosocial support refers to other activities that aim to promote general psychosocial well-being. For example, listening, counselling, lecture, recreation, group activities, and relaxation techniques such as massage and foot bathing. These are typically offered by education, healthcare, and welfare personnel, or lay-volunteers. These activities largely overlap and lie along a continuum, and complement each other. However, it is important that the care providers are aware of their roles and position on this continuum.

3) Results of Round 2

In this round, 88.4% of the respondents rated the modified definition ≥ 4 for its appropriateness, which fulfilled the consensus criterion. The comments from the 9 participants in this round comprised 9 units, with 4 units on definition in general, 2 units on mental health services, 2 units on psychosocial support, and 1 unit in the ‘others’ category. There was an 88.9% agreement between the two researchers on the sub-categories in Round 2.

**a. Definition in general: **Similar to that in Round 1, **Cautions in operation** emerged, including comments such as: ‘Indeed the categorisation of mental health services and psychosocial support is good. However, needs assessment of individuals, and coordinated care of mental health services and psychosocial support are important’. (Profession and position unknown) ‘In the field, the mental health care team held community-salon-meetings, such as tea meetings. In the affected area, there was no human resource to hold such meetings where needed. Such an activity was considered to be a mental health and welfare activity’. (Profession and position unknown) **Alternative categorisation** of care components included comments such as: ‘For me, it is classified as psychiatry, mental health, and life’. (Psychiatrist, dispatched) There was one comment that was not related to the definition, thus this was categorised as **others**.

**b. Mental health services**: These comments emphasized on the importance of mental welfare services and awareness-raising as measures of primary prevention. These comments were sub-categorised as **Others.**

**c. Psychosocial support:** **Raising concerns about the terms** emerged as a sub-category, which included the following comments: ‘The term psychosocial support contains professional nuances. However, simpler wording is needed depending on situations’. (Psychiatrist, local) ‘“Well-being” in the term “psychosocial well-being” needs to be clearly explained or expressed in natural Japanese’. (Profession unknown, dispatched)

**d. Others**: This category included miscellaneous comments such as those stating that the explanation was difficult to understand.

## Discussion

This survey asked participants to rate the appropriateness of the definition of ‘Kokoro-no care’, among mental health and community health care staffs who engaged in the care activities after the Great East Japan Earthquake. The consensus criterion was fulfilled in Round 2, and we adopted the modified definition as final version.

Majority of the participants of this survey were mental health professionals, and engaged in psychiatric or mental health services in daily practice. The teams were dispatched to the affected areas, and their primary mission was to offer mental health services in the early phase of the disaster. With such professional backgrounds and situations in mind, below is a discussion on the context of the comments for each category.

**Definition in general**

In this category, most of the comments referred to the relationship between care components, as seen in the** Continuum of MHSPSS**. The participants in this survey often voiced the importance of the continuum of MHSPSS, as was raised before 4. This point was ever more evident, presumably, in line with the recent trend that traumatic reaction is viewed as on a spectrum, in that traumatic reaction is considered to be a normal reaction to abnormal situations, and posttraumatic stress disorder (PTSD) is seen as a pathological state in which the recovery process from the traumatic reaction is hindered 18. Previously, psychological care during disaster exclusively focused on the treatment of PTSD, often reported as a response to the Indonesian tsunami 2. With a better understanding on the mechanism and trajectory of traumatic reactions 19 ^,^ 20, now, care focuses on facilitating the process of recovery. Additionally, as the mental health community is familiar with the concept of resilience, mental health professionals now have more interest in a population approach, which does not focus on specific risk populations with a pathological traumatic reaction, rather reaches out to the general population, to raise awareness regarding health problems, including non-pathological traumatic reactions, from the public health perspective. Care providers, including mental health professionals, are expected to operate within the continuum to facilitate recovery. With this change in the concept, the role of mental health professionals in disaster response is considered more fluid than before, and this may have affected the comments in the present study.

In the sub-category **Cautions in operation**, the participants addressed that care providers should tend to the affected people flexibly, depending on their situations and needs. Although the mental health professionals in this survey usually engaged in clinical practice at clinics or hospitals, they actively reached out to the affected communities and collaborated with public health professionals during disaster 12. Under the overwhelming situation of a disaster, mental health professionals faced many difficulties of the affected people, which is not merely a medical or mental health problem. These comments may have been drawn from their own experiences and expected roles while tackling the aftermath of the disaster, pointing out that carers have to optimise their care approach, such that it ranges from non-specific intervention to treatment intervention for the people affected by the disaster.

In the category **Alternative categorisation of care components**, the participants expressed a concern that the integrated concept does not appropriately represent ‘Kokoro-no care’. The proposed definition was ambiguous in that it contained different care components, or did not present the real picture of the needed care; thus, causing confusion in care providers and recipients. Their alternative suggestion was that care should be offered based on the needs and situations. This was in line with the above-mentioned **Cautions in operation**. The comments in the sub-category **Whether the care component should be categorised according to the professional involved** indicated that it is difficult to separate the continuum of care merely by the professional involved, and that care provided was unrelated to the profession of the carer. This also suggests the importance of scrutinizing the care component, instead of the professional background.

In the sub-category **Ambiguous use of psychology**, some comments pointed out that the people affected by disaster did not expect the expertise of psychologists in psychosocial care. Psychological First Aid (PFA) 21 is now a standard approach in the acute phase of a disaster; however, the content of the PFA does not require special skills of psychologists, and is a nonspecific humanitarian approach. In approaching the people distressed during a disaster, humane care was recommended. However, the term ‘psychological’ first aid suggests the nuance of expertized skills, thus causing confusion. The comments questioned the incoherence between the concept of ‘psychological first aid’ and what people, both care providers and recipients, really expected from psychological care.

**Mental health services**

**** Under** More specification within mental health services**, there were comments that stressed on specifying the service components provided as mental health services. In the aftermath of the Great East Japan Earthquake, local psychiatric institutions did not function because some of them were inundated or had to evacuate due to the nuclear plant accident 22 ^,^ 23. Under this situation, the care priority of the mental health care teams was to respond to psychiatric needs in psychiatric institutions, rather than to provide psychosocial care in the affected communities. Within this context, some pointed out the importance of explicitly addressing the care components of mental health services, such as psychiatric rescue, psychiatric service, mental health activities, and psychological support. This view was echoed in the comments in this sub-category. Some suggested that, because mental health services differ from psychosocial support, a different term should be used, to avoid confusion among the affected people and care providers. The comment indicated that the explicit description of the role and care components would reduce such confusion. These comments may have been raised because majority of the respondents were mental health professionals, and they stressed on their profession.

**Psychosocial support**

As discussed earlier, regarding psychosocial support, there were many comments on **Whether the care component should be categorised according to the professional involved** in Round 1. In addition, some expressed comments **Raising concerns about the term** in Round 2. Some respondents suggested that the term ‘well-being’ in the proposed definition, was ambiguous. The WHO defines health as ‘a state of complete physical, mental, and social well-being, and not merely the absence of disease or infirmity’^24^. This definition is well accepted in Japan; however, ‘well-being’ is represented by a Katakana character, which is used when there is no appropriate Japanese word for a foreign word. Although familiar, the term, ‘well-being’ has not been well defined. Indeed, it is a multi-dimensional concept that typically involves positive emotions, engagement, and meaning 25. Thus, the meaning of ‘well-being’ should be examined in the context of disasters. ‘Psychosocial well-being’ means a good state including, but not limited to self-realization, work, and contribution to society 7. To achieve psychosocial well-being, the psychological approach is an important avenue, but this is not addressed only by psychological professionals. To achieve psychological well-being, a holistic approach is required; however, the word psychology tends to be related to the related professions, which may have caused the confusion.

**Limitations**

This survey had several limitations. First, majority of the participants were licensed mental health professionals, and usually worked in mental health services, both during disasters and otherwise. The active players in psychosocial support, such as lay-volunteers, social service personnel, and community people, were not included in this survey. Therefore, these comments have to be interpreted with the caution that they are representative of the collective experience of mental health professionals.

Next, feedback comments after Round 1 may have affected the successive rating, which is the nature of the Delphi process. During the process, we found that the comments in Round 1 facilitated brainstorming on the nature and practical cautions of ‘Kokoro-no care’ among the participants, under anonymity. However, we strived to include a wide range of comments, without ignoring minority comment.

The Delphi process was adopted to build consensus, but in fact, we found it useful to gather free ideas based on the participants’ experiences. Such collective thoughts based on the experience of the Great East Japan Earthquake will benefit the process of preparing for and responding to future disasters.

## Competing Interests

The authors have declared that no competing interests exist.
